# Impact of an Innovative Approach to Prevent Mother-to-Child Transmission of HIV — Malawi, July 2011–September 2012

**Published:** 2013-03-01

**Authors:** Frank Chimbwandira, Eustice Mhango, Simon Makombe, Dalitso Midiani, Charles Mwansambo, Joseph Njala, Zengani Chirwa, Andreas Jahn, Erik Schouten, B. Ryan Phelps, Anna Gieselman, Charles B. Holmes, Alice Maida, Sundeep Gupta, Beth A. Tippett Barr, Surbhi Modi, Helen Dale, John Aberle-Grasse, Margarett Davis, David Bell, James Houston

**Affiliations:** Dept of HIV and AIDS; Malawi Ministry of Health; Dept for Global Health, International Training and Education Center for Health, Univ of Washington, and Dept of HIV and AIDS, Malawi Ministry of Health; Management Sciences for Health; Office of HIV/AIDS, US Agency for International Development; Office of the US Global AIDS Coordinator, US Dept of State; Center for Global Health, CDC-Malawi; Div of Global HIV/AIDS, Center for Global Health; EIS Officer, CDC

Antiretroviral medications can reduce rates of mother-to-child transmission of human immunodeficiency virus (HIV) to less than 5% (1). However, in 2011, only 57% of HIV-infected pregnant women in low- and middle-income countries received a World Health Organization (WHO)–recommended regimen for prevention of mother-to-child transmission (PMTCT), and an estimated 300,000 infants acquired HIV infection from their mothers in sub-Saharan Africa; 15,700 (5.2%) of these infants were born in Malawi (2). An important barrier to PMTCT in Malawi is the limited laboratory capacity for CD4 cell count, which is recommended by WHO to determine which antiretroviral medications to start (3). In the third quarter of 2011, the Malawi Ministry of Health (MOH) implemented an innovative approach (called “Option B+”), in which all HIV-infected pregnant and breastfeeding women are eligible for lifelong antiretroviral therapy (ART) regardless of CD4 count (4). Since that time, several countries (including Rwanda, Uganda, and Haiti) have adopted the Option B+ policy, and WHO was prompted to release a technical update in April 2012 describing the advantages and challenges of this approach as well as the need to evaluate country experiences with Option B+ (5). Using data collected through routine program supervision, this report is the first to summarize Malawi’s experience implementing Option B+ under the direction of the MOH and supported by the Office of the Global AIDS Coordinator (OGAC) through the President’s Emergency Plan for AIDS Relief (PEPFAR). In Malawi, the number of pregnant and breastfeeding women started on ART per quarter increased by 748%, from 1,257 in the second quarter of 2011 (before Option B+ implementation) to 10,663 in the third quarter of 2012 (1 year after implementation). Of the 2,949 women who started ART under Option B+ in the third quarter of 2011 and did not transfer care, 2,267 (77%) continue to receive ART at 12 months; this retention rate is similar to the rate for all adults in the national program. Option B+ is an important innovation that could accelerate progress in Malawi and other countries toward the goal of eliminating mother-to-child transmission of HIV worldwide.

Antiretroviral medications can be provided to improve a patient’s own health, prevent vertical HIV transmission from mother to infant, and/or prevent horizontal transmission to an uninfected sex partner. In most resource-limited settings, ART eligibility is based on CD4 cell count or clinical staging. For pregnant women with CD4 ≤350 cells/mm^3^ or at WHO clinical stage 3 or 4, the 2010 WHO PMTCT recommendations include lifelong ART. For HIV-infected pregnant women not eligible for ART, either of two prophylaxis options (called “Option A” and “Option B”) is recommended. Option A involves prophylaxis with a single drug, zidovudine (AZT), during pregnancy, and additional antiretroviral medications during labor, delivery, and the postpartum period. Option B involves triple-drug ART during pregnancy and breastfeeding. Both options include additional antiretroviral medications for infants (1).

In Malawi, the MOH determined the health sector did not have the laboratory and infrastructure capacity to provide universal access to CD4 cell count testing needed to successfully implement either of the two recommended options. Instead, they proposed a modified Option B (called “Option B+”), in which all confirmed HIV-infected pregnant and breastfeeding women are offered lifelong ART regardless of CD4 count or clinical stage. This policy streamlined the process of ART initiation and had the potential to improve maternal health, facilitate access to PMTCT and ART, reduce HIV transmission risk to uninfected male partners, and provide protection against vertical HIV transmission in future pregnancies (4,6). Implementation of Option B+ also required integration of ART into all antenatal care (ANC) settings, training of nearly all health-care workers in a new integrated curriculum, and a change in the adult first-line ART regimen to one that included the antiretroviral medication efavirenz.* Implementation was facilitated by existing task-shifting policies that allow clinical officers, medical assistants, and nurses to start ART (4).

Every integrated PMTCT/ART site in Malawi is visited quarterly by members of a nationally coordinated supervision team composed of MOH service providers, supervisors, supporting partners, and CDC-Malawi staff. Direct supervision of every site in every quarter is the key feature of the national HIV program. Innovative patient registers have been created to permit longitudinal follow-up and cohort analyses for patients receiving antenatal and HIV care. Data collected during these supervision visits include the number of persons started on ART, the reason for starting ART, and, of those started on ART in previous quarters, the number of patients retained in care. These facility-level aggregated data are returned to the central-level MOH, entered into a database, cleaned, and then analyzed to produce MOH’s *Quarterly HIV Programme Reports*,† on which this report is based.

Implementation of Option B+ required training of 4,839 health-care workers and resulted in decentralization of ART to all health centers with ANC, with an increase from 303 ART sites in June 2011 to 641 integrated PMTCT/ART sites in September 2012 (Figure 1). After implementation of Option B+ began in July 2011, the total number of all persons started on ART per quarter increased by 61%, from 18,442 in the second quarter of 2011 to 29,707 in the third quarter 2012.

Implementation of Option B+ resulted in a 748% increase in the number of pregnant and breastfeeding women starting ART, from 1,257 in the second quarter of 2011 (representing 5% of all new ART initiations) to 10,663 in the third quarter of 2012 (35% of all new ART initiations) (Figure 2).

Of the women starting ART in the third quarter of 2011 (the first quarter of Option B+ implementation) who did not transfer care during follow up, 77% continue to receive ART at 12 months (Figure 3). This rate is similar to the 80% 12-month ART retention rate observed among adults who initiated ART in the second quarter of 2011 (the last quarter before Option B+ implementation).

## Editorial Note

In June 2011, PEPFAR (under the leadership of OGAC) and the Joint United Nations Program on HIV/AIDS launched a global plan to virtually eliminate mother-to-child transmission of HIV with the goal of reducing new HIV infections in children by 90% by 2015.§ In Malawi, under the new policy, the number of pregnant and breastfeeding women started on ART has increased and the retention rate has remained similar to the rate for adults continuing to receive ART at 12 months before Option B+ implementation. Option B+ is an important innovation that could accelerate progress in Malawi and other countries toward the goal of eliminating mother-to-child transmission of HIV worldwide.

Barriers to ART provision for pregnant women in resource-limited settings include the need for CD4 cell count, distance between ANC sites where HIV diagnosis is made and ART sites where treatment is started, transportation costs, and human resource constraints that lead to long waiting times and scheduling difficulties (3). The removal of the barrier of CD4 cell count, decentralization of ART into all ANC sites, and the training of nearly all nurses and clinical officers on the new integrated PMTCT/ART guidelines facilitated the increase in the number of pregnant and breastfeeding women started on ART. Implementation of Option B+ in Malawi enabled women to receive ART and ANC services in the same clinic and from the same provider without adversely affecting retention in care.

The seven-fold increase in the number of pregnant and breastfeeding women started on ART per quarter during the first year of Option B+ has multiple potential benefits to mothers, their partners, and their children. For women, ART provides protection for their own health and, therefore, with expansion of ART coverage, a substantial reduction in mortality through the postpartum period can be expected (7,8). For HIV-uninfected sexual partners, ART offers protection from HIV transmission. In Malawi, one third of HIV-infected women are estimated to be in stable relationships with HIV-uninfected partners; studies suggest a substantial reduction in HIV transmission within these relationships in the setting of effective ART (6,9). For children of current and future pregnancies, ART provides protection from HIV infection during pregnancy and breastfeeding. The mother-to-child transmission rate for women on ART is expected to be reduced, from approximately 40% without intervention to less than 5%. With PEPFAR funding, CDC is supporting a nationally representative prospective evaluation to estimate the mother-to-child transmission rate in Malawi (1).

Important challenges and questions remain. Evaluations to assess the cost-effectiveness of this approach are needed, and although 12-month retention rates are reassuring, lifelong ART adherence will need to be maintained. Although high-quality HIV testing is accepted by nearly all women at ANC in resource-limited settings, the Malawi MOH estimates that failure to ascertain maternal HIV status at ANC is now responsible for 54% of new infant infections in Malawi, likely as a result of irregular availability of test kits and poor quality assurance of rapid testing at ANC sites. (10; Frank Chimbwandira, Malawi MOH, personal communication, 2012). With PEPFAR funding, CDC is supporting birth defects surveillance in Malawi and elsewhere because limited data currently are available on the possible adverse effects of efavirenz-based ART regimens on infants exposed in early gestation (5).

The success of Option B+ in increasing ART coverage demonstrates the combined effect of streamlined ART initiation, decentralized and integrated service delivery, policy changes to allow nurses to start ART, and direct supervision of every site. Continued progress in Malawi demands consistent provision of high-quality HIV testing in ANC and continuing efforts to ensure lifelong ART adherence among women started on ART through Option B+.

What is already known on this topic?

Mother-to-child transmission of human immunodeficiency virus (HIV) can be reduced to less than 5% with antiretroviral medications. However many HIV-infected pregnant and breastfeeding women in sub-Saharan Africa still do not receive services to prevent transmission to their infants. An important barrier is the limited laboratory capacity for CD4 cell count, which is recommended by the World Health Organization to determine which antiretroviral medications to start in pregnant and breastfeeding women.

What is added by this report?

In 2011, Malawi implemented a new policy (Option B+) to provide all HIV-infected pregnant and breastfeeding women with lifelong antiretroviral therapy (ART) regardless of CD4 count. The number of pregnant and breastfeeding women started on ART increased by 748%, from 1,257 in the second quarter of 2011 (before Option B+ implementation) to 10,663 in the third quarter of 2012 (1 year after implementation).

What are the implications for public health practice?

Option B+ is an important innovation that could accelerate progress in Malawi and other countries toward the goal of eliminating mother-to-child transmission of HIV worldwide.

## Figures and Tables

**FIGURE 1:**
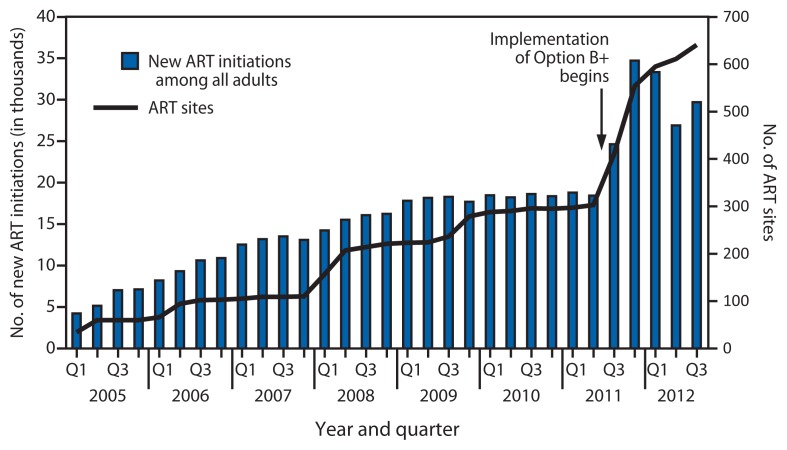
Number of new antiretroviral treatment (ART) initiations among all adults and number of ART sites, by year and quarter — Malawi, 2005–2012

**FIGURE 2:**
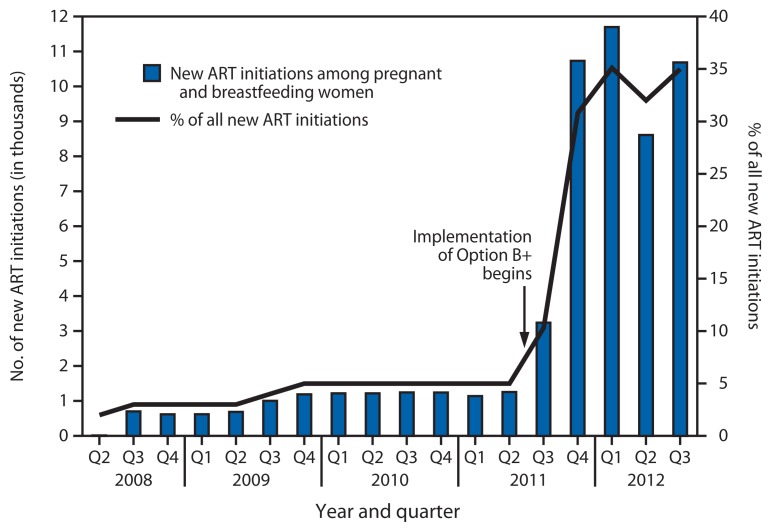
Number of new antiretroviral treatment (ART) initiations among pregnant and breastfeeding women, and percentage of all new ART initiations attributed to this population — Malawi, 2008–2012

**FIGURE 3:**
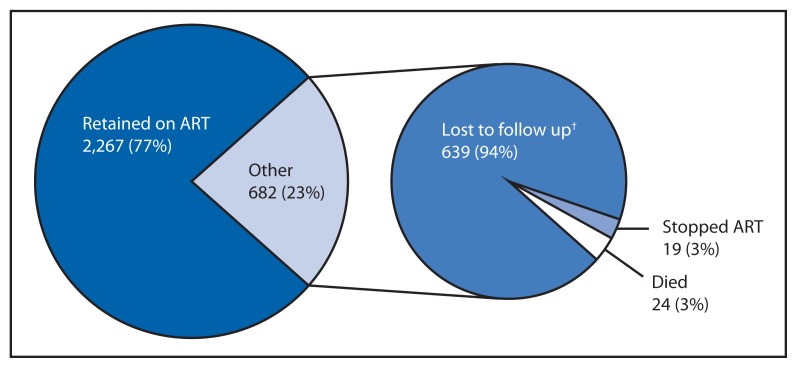
Twelve-month outcomes for women initiating antiretroviral treatment (ART)^*^ — Malawi, third quarter of 2011 ^*^ N = 2,949. A total of 3,241 women initiated ART in the third quarter of 2011. However, 315 women were excluded from this analysis because they were documented to have transferred care from the clinic where they were initiated on ART and outcomes could not be verified. An additional 23 women were excluded because of incomplete information regarding the reason for starting ART. ^†^ Some women labeled as lost to follow up might be deceased 12 months after initiating ART.

## Footnotes

* The MOH chose to use efavirenz as a part of its first-line regimen for all adults, including pregnant and breastfeeding women, because a growing body of evidence indicates that efavirenz has superior efficacy and tolerability compared with nevirapine. Although concerns exist that efavirenz is potentially teratogenic in the first trimester based on animal models, the limited data available on the use of efavirenz during pregnancy do not demonstrate any adverse effect. Additional information on use of efavirenz in pregnancy is available at http://whqlibdoc.who.int/publications/2012/9789241503792_eng.pdf.

† Available at http://www.hivunitmohmw.org/Main/AntiretroviralTherapy.

§ Additional information available at http://www.unaids.org/en/media/unaids/contentassets/documents/unaidspublication/2011/20110609_jc2137_global-plan-elimination-hiv-children_en.pdf.
